# P02.154. Stress management and resilience training among Department of Medicine faculty: a pilot randomized clinical trial

**DOI:** 10.1186/1472-6882-12-S1-P210

**Published:** 2012-06-12

**Authors:** A Sood, K Prasad, V Sharma, D Schroeder, P Varkey

**Affiliations:** 1 Mayo Clinic Rochester, Rochester, USA

## Purpose

Physician distress is common and related to numerous factors including loss of control over the practice environment, workload, specialty choice, experience with suffering, interpersonal relationships, debt, poor self-care, maladaptive coping strategies and stressful life events such as divorce. The current study was designed to assess the effect of a Stress Management and Resiliency Training (SMART) program, for increasing resiliency and quality of life, and decreasing stress and anxiety among Department of Medicine (DOM) Physicians at Mayo Clinic Rochester.

## Methods

Forty DOM physicians at Mayo Clinic Rochester were randomized in a single-blind wait-list controlled clinical trial to either the SMART intervention or a control group for twelve weeks. The intervention involved a single 90 minute one-on-one training in the SMART program. Primary outcome measures assessed at baseline and week 12 included the Connor Davidson Resilience Scale (CDRS), Perceived Stress Scale (PSS), Smith Anxiety Scale (SAS), and Linear Analog Self Assessment Scale (LASA).

## Results

Thirty-two physicians completed the study. A statistically significant improvement in resilience, perceived stress, anxiety and overall quality of life at 12-weeks was observed in the study arm compared to the wait-list control arm (CDRS: +9.8±9.6 vs. -0.8±8.2, p=0.003), (PSS: -5.4±8.1 vs. 12.8±6.6, p=0.010), (SAS: -11.8±12.3 vs. +2.9±8.9, p=0.001) and LASA (+0.4±1.4 vs. -0.6±1.0, p=0.029). No significant difference in any of these measures was noted in the control group.

## Conclusion

This study demonstrates that a single session to enhance resilience and decrease stress among physicians is feasible. Further, the intervention provided statistically significant and clinically meaningful improvement in resilience, stress, anxiety and overall quality of life in the active arm compared to the control group. Future larger clinical trials and wider dissemination of this intervention are warranted.

